# Supplementary material for: The effects of genital myiasis on the diversity of the vaginal microbiota in female Bactrian camels
Source: BMC Vet Res. 2022 Mar 5;18:87. doi: 10.1186/s12917-022-03189-5 (PMC8897907; doi:10.1186/s12917-022-03189-5)
Supplement: Supplementary file 5 — Additional file 5. [file 12917_2022_3189_MOESM5_ESM.zip › MPL201709200_16s_yy/Treat1/css/js_css/jsPDF-master.zip!/jsPDF-master/libs/Downloadify/test.html]

Downloadify


# Downloadify Example

More info available at the Github Project Page

Filename

File Contents  

Whatever you put in this text box will be downloaded and saved in the file. If you leave it blank, no file will be downloaded

You must have Flash 10 installed to download this file.

## Downloadify Invoke Script For This Page

```
Downloadify.create('downloadify',{
  filename: function(){
    return document.getElementById('filename').value;
  },
  data: function(){ 
    return document.getElementById('data').value;
  },
  onComplete: function(){ 
    alert('Your File Has Been Saved!'); 
  },
  onCancel: function(){ 
    alert('You have cancelled the saving of this file.');
  },
  onError: function(){ 
    alert('You must put something in the File Contents or there will be nothing to save!'); 
  },
  swf: 'media/downloadify.swf',
  downloadImage: 'images/download.png',
  width: 100,
  height: 30,
  transparent: true,
  append: false
});
```
